# Elevated expression of *ANTXR1* gene in tumors is a poor prognostic biomarker for patients with bladder cancer

**DOI:** 10.3389/fmolb.2024.1520223

**Published:** 2025-01-23

**Authors:** L. S. Franco, S. Arunachalam, A. Chauhan, S. A. Kareff, P. L. Hallenbeck

**Affiliations:** ^1^ Seneca Therapeutics, Inc., Blue Bell, PA, United States; ^2^ Sylvester Comprehensive Cancer Center, University of Miami Health System, Miami, FL, United States; ^3^ Lynn Cancer Institute, Boca Raton Regional Hospital, Boca Raton, FL, United States

**Keywords:** TEM8, prognostic biomarker, bladder cancer, downstream pathway, survival

## Abstract

The TEM8 protein coded by the *ANTXR1* gene represents an emerging biomarker in solid tumors. In addition to the various roles TEM8 plays in oncogenesis, including angiogenesis, epithelial-to-mesenchymal transition, and cell migration, it has also been shown that the overexpression of the *ANTXR1* gene in solid tumors correlates with poor prognostic indicators in several solid tumor histologies. As such, TEM8 has been identified as the target of novel oncologic therapies. It is especially attractive given its selective expression on the surface of solid tumor cells and associated stromal cells, such as cancer stem cells, invasive cancer cells, and immune cells, such as macrophages, angiogenic endothelial cells, pericytes, and cancer-associated fibroblasts. Furthermore, TEM8 plays this unique role as a mostly non-mutated gene in solid cancers. Here, we demonstrate that elevated expression of ANTXR1 in bladder cancer showed a statistical difference not only in overall survival (OS) but in progression-free survival (PFS), confirming the prognostic biomarker power of *ANTXR1* expression.

## Introduction

The tumor endothelial marker 8 (TEM8) or anthrax receptor 1 (ANTXR1) is a protein that is encoded by the *ANTXR1* gene and is part of the receptors that are involved in the facilitation of the entry of anthrax into the cell (Alcalá et al., 2019). It is homologous to the protein capillary morphogenesis protein 2 (CMG2 or ANTXR2), that the anthrax toxin binds to. However, the severity of pathology from the toxin is very different. CMG2 is the major receptor involved in the lethality of the anthrax toxin *in vivo* (Fu, et al., 2010). TEM8, on the other hand, is expressed during angiogenesis, as well as in some somatic tissues and human tumors (Friebe et al., 2015). It was thought that higher expression of TEM8 in tumors was mainly due to angiogenesis within the tumor, however, high TEM8 expression has also been observed in stroma cells like cancer associated fibroblasts (CAFs) and pericytes (Hsu et al., 2022; Kareff et al., 2023) and cancer cells themselves (Hoye, et al., 2018).

TEM8 has been shown to activate different pathways in tumors like that of PI3K/Akt/mTOR in gastric cancer (Cai, et al., 2020) and glioblastoma (Kundu, et al., 2024). It has also been linked with the activation of the Wnt/beta catenin pathway in lung adenocarcinoma (Ding, et al., 2021) and in recurrent glioblastomas (Kundu, et al., 2024). The TGF-beta pathway, one of the most common pathways involved in cancer, is also activated in gastric cancer and is correlated with high expression of *ANTXR1* (Huang et al., 2020). Lastly, it is known that TEM8 is involved in the collagen pathway by facilitating the uptake of collagen VI in cancer stroma, which is key for cancer survival (Hsu, et al., 2022), and in breast cancer, in which Chen, et al. demonstrated that it was involved in collagen trafficking (Chen et al., 2013).

TEM8 has been targeted before due to its correlation with the pathways that are involved in tumor progression, invasion and aggressiveness. There have been several reports involving monoclonal antibodies that are bound to TEM8 and showed slowed tumor growth in animal models (Chaudhary et al., 2012).

Seneca Valley Virus (SVV) is a naturally occurring swine picornavirus that is spread throughout the world that does not infect humans (Venkataraman, et al., 2008). SVV does, however, infect solid cancer cells. The initial isolate called SVV-001 has been studied in three prior clinical trials when systemically delivered to patients with solid cancers and demonstrated promising signals of efficacy and safety (Rudin, et al., 2011). The receptor of SVV was identified to be TEM8 (Evans, et al., 2018; Corbett et al., 2022). It has also been demonstrated that SVV-001 infection in solid tumors can induce an influx of immune cells by turning the tumor microenvironment from “cold” (low immunogenicity) to a “hot” tumor (high immunogenicity), thus enabling synergistic checkpoint inhibitor function. SVV has high specificity for TEM8 expressing tumors, making SVV a promising candidate for difficult to treat solid cancers that express TEM8.

More ways to possibly predict the outcome of a patient are being investigated. Currently, many cancer types rely on the TNM classification, but this system does not take into account the tumor microenvironment, which plays a key role in tumor progression (Wang, et al., 2023; Luo et al., 2021). An alternative way that has been explored to predict outcome is the Estimation of STromal and Immune cells in MAlignant Tumors using Expression (ESTIMATE) data’ that uses tumoral gene expression signatures to determine the number of stromal cells and infiltration of immune cells in the tumor (Yoshihara, et al., 2013). In gastric cancer, the stromal score was demonstrated to be related to immune gene signature and macrophage infiltration (Wei et al., 2020). Similarly, in colorectal cancer the combination of the TNM staging system in combination with the stromal and immune score gives a better accuracy than the TNM alone (Luo et al., 2021). Other prognostic biomarkers are needed to predict with a higher accuracy a patient’s outcome in different types of cancer.

In previous studies, TEM8 has been described as a potential biomarker for different types of cancer including colorectal cancer, where TEM8 expression was significantly correlated with TMN stage and high TEM8 expression in serum was correlated with worse overall survival than those with lower TEM8 expression (Pietrzyk et al., 2021). Similarly, TEM8 was described as a new biomarker in pancreatic cancer (Alcalá et al., 2019) and for gastric cancer, in which it was suggested as a prognostic biomarker due to its relationship with the immune and stromal scores as well as correlation between high expression with clinicopathological parameters (Huang et al., 2020; Cai, et al., 2020). In lung cancer, higher TEM8 expression was associated with lower OS (Sun, et al., 2021).

Bladder cancer is the most common urogenital cancer (10th most prevalent), affecting more than 1.6 million people worldwide (Castaneda et al., 2023; Flores Monar et al., 2023). This cancer is one of the most onerous cancers, necessitating personal healthcare costs. In 2015 in the U.S., bladder cancer incurred a cost of $7.93 billion and is expected to ∼ double by 2030 (Flores Monar et al., 2023). More biomarkers (predictive and/or prognostic) are needed to manage patients with this cancer. Currently, there are only six biomarker tests approved by the FDA for surveillance for bladder cancer, but many of them still present high false-positive rates (Flores Monar et al., 2023). Some of the molecular biomarkers seem promising in early development, however they have yet to successfully transition to the bedside (Castaneda et al., 2023). Here, we studied the effect of *ANTXR1* gene expression in different types of cancers and chose bladder cancer to understand the implications of high expression of *ANTXR1* as a poor prognostic marker. We also investigated the pathways that are enriched in tumors downstream of the *ANTXR1* gene to complement the effect of this gene in the tumor metabolism.

## Materials and methods

### Overall survival and progression-free survival

The Cancer Genome Atlas was used to study the expression of *ANTXR1* and its prognosis. The clinical, RNA expression data are downloaded from cbioportal (https://www.cbioportal.org).

Multiple cancer types were analyzed. The upper quartile of TEM8 fragments per kilobase per million mapped reads (FPKM) was labeled as high expression, and the rest of quartiles are labeled as low expression of *ANTXR1*. The Kaplan-Meier method was used to estimate overall survival as a function of time. Survival statistical differences were assessed by Log-rank test with a threshold of p value ≤ 0.05. The Kaplan-Meier analysis is done using KaplanMeierFitter.

For bladder cancer specifically, TCGA data had 133 patients from which 86 patients were treated with chemotherapy, 18 patients were treated with radiotherapy, 16 patients were treated with a combination of chemotherapy and radiotherapy, and 13 patients were treated with other treatments (Supplementary File S1).

For melanoma, the TCGA data obtained was from the Pan cancer atlas data (448 samples). Other data for the calculation of the immune score of melanomas was obtained from immunogenic studies made in DFCI (metastatic melanoma, 110 samples) and UCLA (metastatic melanoma, 38 samples). A graphical description of the workflow of the study is represented in Supplementary Figure S1.

### Stromal score and immune score

Stromal and immune score were calculated using algorithms like ESTIMATE (Estimation of STromal and Immune cells in MAlignant Tumor tissues using Expression data) which analyze gene expression profiles to determine the relative proportion of these cell types within a tumor. For the stromal score, 141 genes were analyzed using ssGSEA as a stromal signature in tumors and for the immune score, 141 genes were analyzed using ssGSEA as a immune signature, as described elsewhere without modifications (Yoshihara, et al., 2013). Statistical differences in the high and low expressing *ANTXR1* populations were assessed using the Wilcoxon-test, using a threshold of p value ≤ 0.05.

### Validation of membrane bound biomarkers in bladder cancer

We identified 28 previously published survival associated individual genes (*HRAS, SPP1, VEGFA, TGFB1, CDKN2A, FGF3, KRT38, FABP1, GUCA1C, PYGM, CLEC3B, KCNB1, FBXL22, CFD, HLF, FLT1, KDR, CXCR7, FGFR3, TACSTD2, ABCB1, ERBB2, ITGB8, MACC1, SPON2, RHOA, CXCR4, CXCL12*). Each of these biomarker candidates were investigated using TCGA cancer patients for prognosis (overall survival and progression-free survival) and compared with *ANTXR1* expression levels and patient prognosis. The number of patients analyzed for the *CXCR7* was 406 for OS and 174 for PFS. The number of patients analyzed for the *SPON2* was 406 for OS and 174 for PFS.

*Deseq2 and GSEA:* Differential expression analysis (DEA) with TCGA RNA data was done using PyDESeq2 (https://pydeseq2.readthedocs.io/en/latest/index.html) version DESeq2 (v1.34.0) (For more details: https://github.com/owkin/PyDESeq2?tab=readme-ov-file#documentation). Gene set enrichment analysis (GSEA) was done using GSEAPY package (https://gseapy.readthedocs.io/en/latest/introduction.html). Gene ontology, Kyoto Encyclopedia of Genes and Genomes analysis, Hallmark and Reactome pathway analysis were performed to identify possible enrichment of genes with specific biological themes. The thresholds for statistical significance were analyzed by the following criteria: log(fold change) > 1 corresponds to upregulated pathway analysis; and log(fold change) < − 1 corresponds to a downregulated pathway analysis. And adjusted p value < 0.05.

## Results

### Stromal score is correlated to *ANTXR1* expression in tumors

Stromal score represents the presence of stromal tissue, and more precisely the number of cells in the tumor microenvironment of a tumor. In this particular study, stromal score was determined by using the stromal signature of 141 genes using single-sample gene set-enrichment analysis (ssGSEA) as described before (Yoshihara, et al., 2013).

**FIGURE 1:**
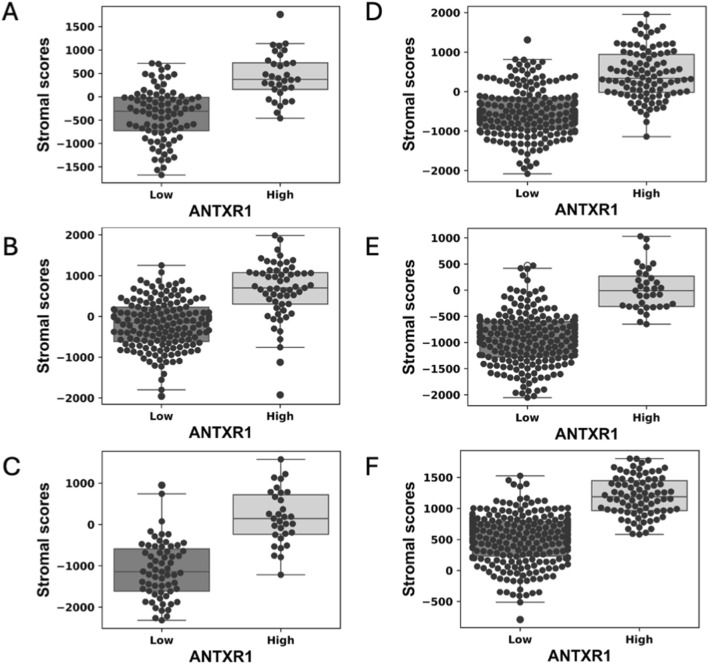
High expression of ANTXR1 in tumor is correlated with high stromal score in different types of cancer. Box plot of stromal score in **(A)** Lung squamous cell carcinoma, **(B)** Lung adenocarcinoma **(C)** Bladder urothelial carcinoma **(D)** Head and neck squamous carcinoma **(E)** Colorectal adenocarcinoma and **(F)** Kidney renal clear cell carcinoma, analyzed by high or low expression of ANTXR1 in tumor. The line in the middle represents the median of the stromal score; top and bottom of the box represent the 75% quartile and 25% quartile of the patients, respectively.

We analyzed eleven different types of cancer for differences of stromal score when there is high or low expression of *ANTXR1* in tumors (basal breast cancer, melanoma, lung squamous cancer, lung adenocarcinoma, colorectal adenocarcinoma, head and neck squamous cell carcinoma, cervical squamous cell carcinoma, bladder urothelial carcinoma, kidney renal clear cell carcinoma, glioblastoma, and metastatic melanoma). Six out of the eleven types of cancer analyzed showed a statistically significant difference of the stromal score when comparing high or low expression levels of *ANTXR1* in each group (Figure 1). The other types of cancer did not show a statistically significant difference (not shown). This figure shows that *ANTXR1* expression has a direct correlation to stromal scores, meaning the higher the expression of *ANTXR1,* the more directly correlated it is to higher stromal scores. This is expected due to the potential expression of *ANTXR1* in different types of stroma cells.

### Immune score may not be correlated to *ANTXR1* expression in tumors

Immune score represents the infiltration of immune cells into the tumor. In this particular study, it was determined by using the immune signature of 141 genes using ssGSEA, as we did with the stromal score (Yoshihara et al., 2013).

**FIGURE 2:**
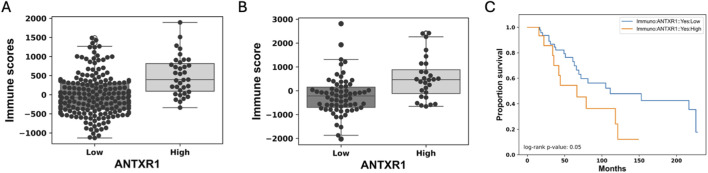
Low expression of ANTXR1 in tumor seems to be correlated with immune response. Box plot of the immune score representing the infiltration of immune cells in **(A)** Colorectal adenocarcinoma, and **(B)** Bladder urothelial carcinoma. The line in the middle represents the median of the immune score; top and bottom of the box represent the 75% quartile and 25% quartile of the patients, respectively. **(C)** Survival curve of patients with Melanoma treated with immunotherapy when there is high (orange) or low expression of ANTXR1 (blue) showing a statistical difference between the two populations.

We analyzed eleven different types of cancer for differences of immune score in varying rates of expression of *ANTXR1* in tumors (same types of cancer as the stromal score). Only two (bladder urothelial cancer and colorectal adenocarcinoma) out of the eleven types of cancer analyzed showed a statistically significant difference of the immune score with different levels of expression of *ANTXR1* (Figure 2). The other types of cancer did not show a statistically significant difference between the high and low *ANTXR1* expression groups (not shown). This figure shows that, paradoxically, a higher immune score (more infiltration of immune cells) is correlated to higher levels of *ANTXR1,* suggesting that for some cancers the infiltration of immune cells may not be a definitive factor when treating cancer. Supporting this suggestion, we also found that in the presence of higher levels of *ANTXR1* in melanoma, a difficult cancer to treat, correlated with poor prognosis in terms of overall survival in patients when treated with immunotherapy.

To investigate the impact of high or low expression of *ANTXR1* in different types of cancer, the probability of survival (overall survival and progression-free survival) was determined for the eleven types of cancer described above as well as small cell lung cancer, for a total of twelve types of cancer.

**FIGURE 3:**
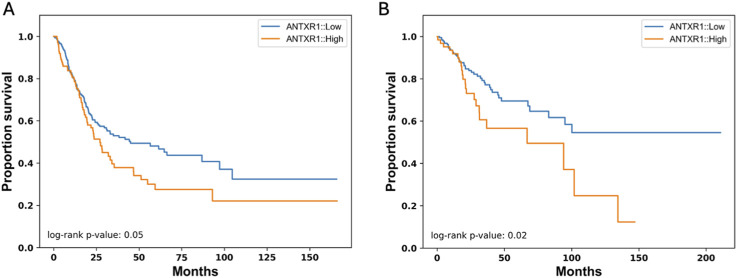
High expression of ANTXR1 is correlated to low overall survival. Kaplan-Meier survival curve for overall survival in **(A)** Bladder cancer and **(B)** Cervical squamous cell carcinoma and endocervical adenocarcinoma when there is high (orange) or low expression of ANTXR1 (blue) showing a statistical difference between the two populations in both types of cancers.

In Figure 3 we demonstrate that the probability of overall survival (OS) for bladder cancer and cervical squamous cell carcinoma is correlated with the tumor expression of *ANTXR1* gene. High *ANTXR1* expression correlates with lower probability of survival *versus* when there is a low expression of *ANTXR1*. Thus, *ANTXR1* expression is a statistically significant biomarker for prognosis in bladder cancer and cervical squamous cell carcinoma.

**FIGURE 4:**
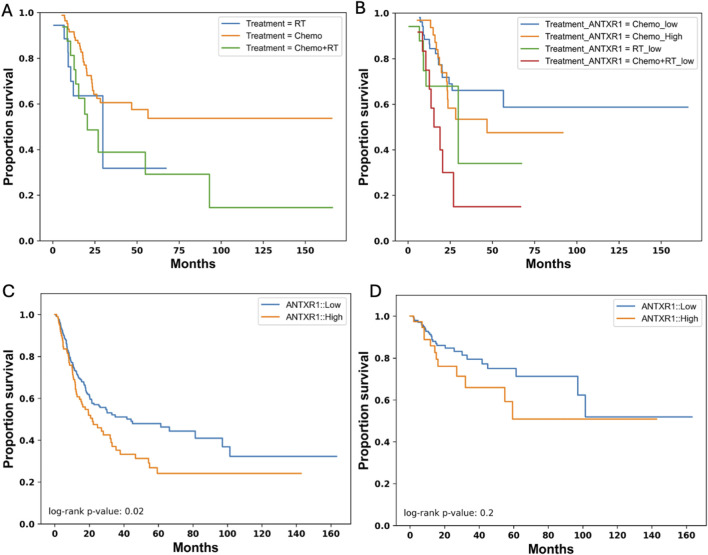
Survival in bladder cancer patients expressing different levels of ANTXR1. Kaplan-Meier survival curve for overall survival in bladder cancer patients separated by **(A)** different treatments (RT: Radiotherapy (blue); Chemo: chemotherapy (orange); Chemo + RT: chemotherapy and radiotherapy (green)); and separated by **(B)** treatments and high or low expression of ANTXR1 in tumor (Chemo_High: chemotherapy with high expression of ANTXR1 (orange); Chemo_low: chemotherapy with low expression of ANTXR1 (blue); RT_low: Radiotherapy with low expression of ANTXR1 (green); Chemo + RT_low: chemotherapy and radiotherapy with low expression of ANTXR1 (red)). Kaplan-Meier survival curve for **(C)** Progression Free survival when there is high (orange) or low expression of ANTXR1 (blue). Kaplan-Meier survival curve for **(D)** Disease-free survival with when there is high (orange) or low expression of ANTXR1 (blue) in tumor.

OS may vary depending on the treatment that the patient received, therefore we analyzed the probability of survival of bladder cancer patients according to the treatments received. Figure 4A shows that patients that are treated with chemotherapy have a higher probability of survival for a longer time (∼0.55) than patients treated with radiotherapy or a combination of both. To understand if *ANTXR1* expression would have any impact on the probability of survival depending on the treatment of the patient, the probability of survival was analyzed according to these parameters. Figure 4B shows that when patients are treated with chemotherapy and have low expression of *ANTXR1,* they have a higher survival probability (∼0.6) for more than 150 months than patients expressing high levels of *ANTXR1* (probability of survival ∼0.5 for 90 months). This trend is similar in patients treated with radiotherapy and the combination of radiotherapy with chemotherapy. This indicates the impact of *ANTXR1* expression independent of the treatment received by the patient.

While OS refers to the time from treatment initiation until death, other measurements like progression-free survival (PFS) and disease-free survival (DFS) can be related in some, but not all types of cancer. PFS is the time from treatment initiation until disease progression and DFS refers to the time from treatment until recurrence of disease. To further investigate the relationship between different types of survival, more specifically PFS and DFS, and expression of *ANTXR1* in bladder cancer patients, we analyzed the probability of survival for bladder cancer patients according to these parameters. Figures 4C, D show the PFS of bladder cancer patients and DFS in cancer patients, respectively. PFS shows congruency between the overall survival and PFS showing a p value less than 0.05. This demonstrates a statistically significant difference between a patient that expresses high levels of *ANTXR1* expression vs low levels of *ANTXR1* expression in bladder cancer. However, the *ANTXR1* expression levels suggest having no impact in the DFS. In conclusion, high expression of *ANTXR1* in bladder cancer is a predictor of bad prognosis even when the patient is using different cancer treatments, and it can be observed in both overall and progression free survival rates.

### *ANTXR1* expression as a unique gene to predict prognosis in bladder cancer

**FIGURE 5:**
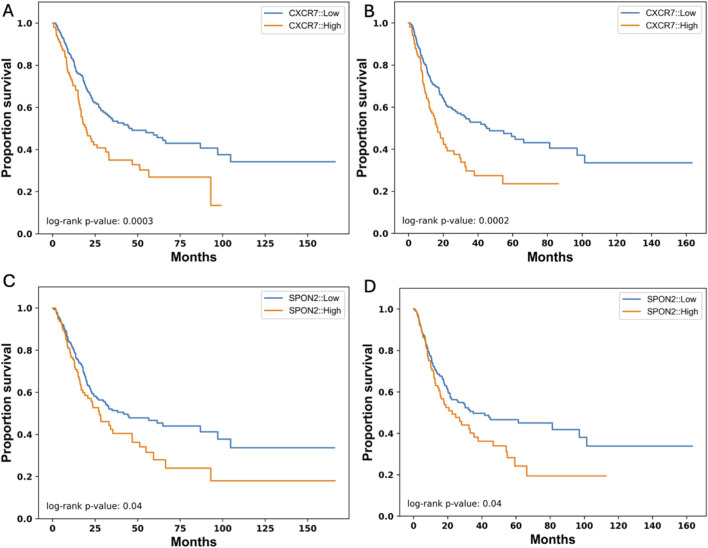
Other Cancer-Associated Membrane Protein genes that their expression affect survival in bladder cancer. Expression of CXCR7 in tumor (high in orange vs. low in blue) affects overall survival **(A)** and progression free survival **(B)** represented by a Kaplan-Meier survival curve. Similarly, expression of SPON2 in tumor (high in orange vs. low in blue) affects overall survival **(C)** and progression free survival **(D)** represented by a Kaplan-Meier survival curve.

In previous studies TEM8, the protein expressed from *ANTXR1*, has been associated with lipid rafts, which are known to have clusters of membrane proteins (Ju et al., 2010). To understand the uniqueness of TEM8 as a membrane protein predictor of good or poor prognosis, we analyzed for low and high expression of 28 different membrane protein encoding genes and examined the probability of survival. Of the twenty-eight genes, only two genes showed a statistically significant difference between high and low expression in terms of overall survival and PFS. These genes correspond to *CXCR7* and *SPON2* (Figure 5). Figures 5A, B show that when there is low expression of *CRCR7* the probability of survival increases in both overall survival and PFS, respectively. The p value in both comparisons show the high impact of *CXCR7* expression in prognosis of bladder cancer patients. For *SPON2*, there is a significant difference in both overall survival and PFS (Figures 5C, D, respectively) when there is low expression vs. high expression of this gene. However, the impact of *SPON2* is lower than the impact of *CXCR7*. In conclusion, after analyzing ∼30 membrane protein genes for their role in survival, only *ANTXR1*, *CXCR7*, and *SPON2* demonstrated a statistically significant impact. Future studies will investigate potential associations involving these proteins. This demonstrates the uniqueness of *ANTXR1* as a prognosis predictor in bladder cancer.

### Pathways associated with high *ANTXR1* expression in bladder cancer

**FIGURE 6:**
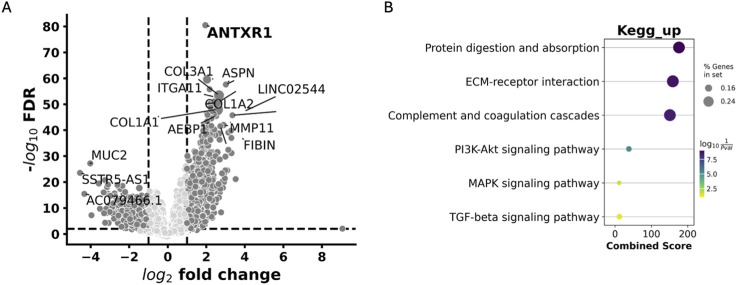
Pathways associated with high expression of ANTXR1 in bladder cancer. **(A)** Volcano plot of Differentially expressed genes impacted by ANTXR1 high expression. **(B)** Gene set enrichment analysis shows KEGG pathways upregulated in ANTXR1 high expression patients. Darker and wider dots represent more genes differentially expressed within the same pathway; lighter and smaller dots represent less genes differentially expressed within the same pathway.

After understanding the impact of *ANTXR1* expression in tumors and its impact on tumor prognosis, we wanted to further investigate the possible mechanisms and/or pathways that were impacted by the expression of *ANTXR1* that might lead to poor prognosis. To understand this, Gene Set Enrichment Analysis (GSEA) was performed on bladder cancer patients with high *ANTXR1* expression (Figure 6). On the volcano plot (Figure 6A) we discerned between the genes that were overexpressed (activated) when *ANTXR1* was highly expressed. These genes were then divided into the pathways in which they were involved (Figure 6B) using KEGG pathway analysis.

The GSEA revealed that among the top 20 KEGG pathways associated with *ANTXR1* expression, the PI3K-Akt signaling pathway, MAPK signaling pathway, and TGF-beta signaling pathway were prominent. These pathways are well-known for their roles in cancer activation and the aggressiveness of the disease. This KEGG analysis was also congruent with MSigDB hallmark pathways (Supplementary Table S1), where TGF-beta signaling is within the top 20 pathways associated among other signaling pathways related to aggressiveness of the tumor such as KRAS, TNFalpha signaling pathways and inflammatory responses (IL-6/STAT3, IL-2/STAT5 and IFNgamma pathways).

## Discussion

TEM8 is part of the tumor endothelial family of proteins and is part of the anthrax receptors, in addition to the capillary morphogenesis gene 2 (CMG2). Although these proteins are homologous, in reality they have quite different roles in terms of disease. Here, we have focused on the role of TEM8, its expression in different types of cancer, and its potential impact in cancer patients.

First, the stromal score was analyzed for different types of cancer evaluated when there was high or low expression of *ANTXR1*. Stromal cells are thought to have important roles in tumor growth, disease progression and drug resistance (Yoshihara, et al., 2013), and tumor related stroma has a role in progression, metastasis of tumor and response to chemotherapy (Stein et al., 2019). The tumor microenvironment (TME) has been demonstrated to play a key role in solid cancers and therefore, the stromal score could be a good indicator of prognosis in solid cancers. This statement has also been tested before to find complementary ways to the long known TMN stage score (Xu et al., 2023). Here, the stromal score in six out of eleven cancer types analyzed, had a significant difference between low and high expression of *ANTXR1*. This implies a reciprocal relationship between *ANTXR1* expression level and stromal score, suggesting that *ANTXR1* could be a potential prognostic indicator. That there is more *ANTXR1* expressed with a greater amount of stroma cells is congruent with the fact that *ANTXR1* expression has been found in some stromal cells including cancer stem cells (Szot et al., 2018).

Another way to predict prognosis in patients with cancer is the immune score, and sometimes this prediction can mirror the stromal score. The immune score corresponds to the expression of key markers of immune cells found in the tumor which represent the immune cells infiltrated with potentially better outcomes. Here, we observed that two of the cancer types analyzed had a significant difference when analyzing immune score in terms of high or low expression of *ANTXR1*. This indicates that, for the majority of the cancer types studied, high stromal scores are correlated with high expression of *ANTXR1 -but* not necessarily the immune score. This finding is similar to those of renal cancer cells, where high levels of CD8^+^ infiltration do not correlate with a favorable prognosis (Xu et al., 2023). Furthermore, this paradox is also present in patients with melanoma in whom the immune score did not reflect any significant difference between high or low expression of *ANTXR1.* However, patients had a longer survival probability (Figure 2C) when patients with low expression of *ANTXR1* were treated with immunotherapy. However, in previous studies high *ANTXR1* expression has been correlated with tumor associated macrophage differentiation and immunosuppression factor in the TME (Huang et al., 2020) suggesting that the immune score might be low. The relationship between immune score and prognosis prediction still needs to be further investigated, as well as its potential relationship with *ANTXR1* expression.

TEM8 has been found in different studies to be a poor prognostic marker especially for a native mostly unmutated gene. Here, we analyzed different types of cancer in terms of probability of OS and expression of *ANTXR1* and found that bladder cancer and cervical squamous cell carcinoma showed significant statistical difference, but there were some cancers (small cell lung cancer OS (p = 0.06) and breast cancer PFS (p = 0.07)) that approached statistical significance. More clinical data analyzed within each cancer type can further clarify the impact of *ANTXR1* expression in these cases. This also translates into the analysis of PFS or DFS, which give another perspective of the prognosis of the patients for different cancers. For bladder cancer, the impact of *ANTXR1* expression showed a statistical difference not only in OS but in PFS, confirming the prognostic biomarker power of *ANTXR1* expression. Similarly, in gastric cancer high ANTXR1 expression was correlated with poor OS and PFS (Huang et al., 2020). It is important to highlight that, to compare between bioinformatic studies of *ANTXR1* expression, it is necessary to understand how the data was analyzed differently in each study, for instance, varying parameters for gene expression.

*ANTXR1* expression is generally associated with tumor metastasis, aggressiveness and tumor invasion in different types of tumors (Hoye et al., 2018). To fully understand the impact of high *ANTXR1* expression, it is key to determine the downstream pathways affected that could potentially lead to tumor progression and aggressiveness. In bladder cancer, the clinical data analyzed via gene set enrichment analysis (GSEA) showed that the extracellular matrix (ECM)- receptor interaction pathway was upregulated, as well as other cancer associated pathways such as PI3K-Akt, MAPK, and TGF-beta signaling pathways (KEGG pathways). These upregulated pathways were also confirmed using the MSigDB Hallmark pathways, including the KRAS Hallmark pathway that is partially in the PI3K-Akt KEGG pathway. One of the key genes in the ECM-receptor interaction pathway is collagen, which has been previously described as a key protein for cancer cell survival mediated by TEM8 in tumor-associated stroma (Hsu et al., 2022) and TEM8 association with the PI3K-Akt signaling pathway in glioblastoma (Kundu et al., 2024) and gastric cancer (Cai et al., 2020). TGF-beta signaling pathway has also been found to be enriched in gastric cancer (Huang et al., 2020), and it is involved with the telomerase (hTERT) enzyme in upregulating the urokinase-type plasminogen activator (uPA) for endothelial-mesenchymal transition (EMT) (Jaiswal and Yadava, 2020; Jaiswal and Yadava, 2019). EMT is one of the principal mechanisms responsible for changes in the ECM and metastasis, indicating poor prognosis in patients (Jaiswal and Yadava, 2020). Since TEM8 also serves as a receptor for uPA via EGFR phosphorylation (Zhang et al., 2018) it should be further investigated the relation between TEM8 and EMT pathways, as this will confirm its role as a prognosis indicator. Regarding the MAPK signaling KEGG pathway, more specifically the p38 MAPK pathway, contains the *NLK* gene that is a precursor for the Wnt pathway, which has been previously demonstrated to be key for metastasis and tumor proliferation in lung adenocarcinoma (Ding et al., 2021). Our findings confirm the unique role of high *ANTXR1* expression in bladder cancer as a precursor for key signaling pathways that induce tumor aggressiveness, metastasis and invasion, indicating a poor prognosis. Future studies need to investigate the role of *ANTXR1* expression in other pathways like EGFR, the complement and coagulation cascades, as well as metastatic pathways including the potential role of TEM8 in the EMT process (with uPA and transgelin), and its importance in tumor biology.

*ANTXR1* expression has been linked to different proteins that may be interacting with TEM8 in the membrane, but to understand its role of tumor prognosis we analyzed the expression level of different membrane proteins and how it was impacting OS and PFS. Of more than 25 membrane proteins, only *CXCR7*, *SPON2* and *ANTXR1* had a significant difference in probability of survival in bladder cancer patients (only ∼10%). *CXCR7* expression has been linked with aggressiveness or metastasis, progression of the tumor and poor overall survival across multiple cancer types (Al-toub et al., 2019). Similarly, high expression of *SPON2* in laryngeal squamous cell carcinoma was associated with metastasis and higher pathological grade (Zhang et al., 2024). These findings and the above confirm the uniqueness of *ANTXR1* as a membrane protein with prognostic biomarker significance.

Although we described a bioinformatic study analyzing clinical data from patients with different types of cancers, further investigations must address not only the *ANTXR1* expression at the RNA, but at the protein level (TEM8). TEM8 has five isoforms that differ mainly in the membrane and intracellular domain and occur after alternative splicing (Vargas et al., 2012). It is still unknown the tropism of expression of these isoforms (cancer or angiogenic cells), or when the isoforms are expressed. There are two indications that the isoforms could play a different role in cancer cells *versus* in somatic cells. First, it was previously found that alpha-smooth muscle actin and transgelin are two intracellular factors that alter the form of TEM8 extracellularly (Yang et al., 2011). As the isoforms have different intracellular lengths, this also may alter the surface form of TEM8, and also potentially affect the downstream pathways that are upregulated in angiogenic and cancer cells. Second, in previous studies it was demonstrated that some post-translational modifications like N-glycosylation of extracellular domain of TEM8 are key for Seneca Valley Virus (SVV) binding to TEM8 in solid cancer cells (Jayawardena et al., 2018); and it is known that this oncolytic virus only infects solid cancer cells (Venkataraman et al., 2008; Rudin et al., 2011). Further studies will explore the role of the isoform in cancer metabolism, as well as the impact that these might have in the downstream pathways from *ANTXR1*.

As TEM8 becomes a more important target for cancer cell killing targeting due to its important role in carcinogenesis as well as selective high expression in solid cancer cells and associated stromal cells, a need to find a specific therapeutic agent to TEM8 has grown over time. Direct pathway inhibitors, RNA targeting, DNA-based vaccines, antibodies, antibody-like molecules, antibody-drug conjugates, CAR-T cells and oncolytic viruses have been used before in pre-clinical stages (Kareff et al., 2023). Most of these approaches are still in the pre-clinical phase. Antibodies and antibody-drug conjugates (ADCs) have demonstrated some success in clinical trials like the monoclonal antibodies SB5 and AF334; a tri-specific killer engager (TriKe) that lead to anti-angiogenic and anti-stroma effects; an ADC with MMAE with high tumor killing and anti-stroma effects; and CAR-T cells that blocked neo-vascularization in breast cancer (Kareff et al., 2023). However, no other therapeutic agent against TEM8 has shown any promising efficacy clinical trials except SVV. This oncolytic RNA virus uses TEM8 to infect TEM8 positive solid cancer cells with high specificity and delivers durable anti-tumor responses by turning “cold” non-immunogenic tumor into a “hot” immunogenic tumor. Further, it has been shown that the treatment of solid cancers in difficult to treat solid tumor animal models with of SVV and immune checkpoint inhibitors can eradicate primary tumors and block secondary tumors from developing indicating that an anti-tumor immune response developed (Corbett et al., 2022).

The role of *ANTXR1* as a prognostic biomarker has been demonstrated not only in this study for bladder cancer, but for other types of cancer. The impact of *ANTXR1* expression may also contribute to the prediction of some therapies’ success such as immune checkpoint inhibitors, TEM8 targeted therapies, or even oncolytic virotherapy. However, this needs to be further investigated as the role of TEM8 in cancer progression is still been elucidated, as well as its effects in other well-known pathways such as TGF-beta, EGFR, MAPK and Wnt-beta catenin. A starting step could be to analyze more real-world data, such as the publicly available in TCGA database, but other available sources could have contributed more valuable information and confirmed our hypothesis in this study. More available data from different sources for these types of studies could speed the advances of the field and specifically for the discovery of the role of TEM8. Nevertheless, future experimental studies would be needed to confirm the bioinformatic analysis done in this study.

In conclusion, in this paper we demonstrated that *ANTXR1* expression has a unique role as a prognostic biomarker not only for bladder cancer, but also for other types of cancer. The downstream activated pathways found in solid cancers with high expression of *ANTXR1* confirmed what has been found in previous studies in other types of cancer., i.e., to be linked with cancer aggressiveness, invasion and metastasis. SVV is the only therapeutic agent with promising efficacy in clinical trials against TEM8 expressing tumors with high specificity. Further studies will address the impact of the *ANTXR1* expression at the protein level, as it could give more information or the role in cancer cells vs. somatic cells.

## Data Availability

Publicly available datasets were analyzed in this study. This data can be found here: https://www.cbioportal.org and in the Supplementary Material.
