# Supplementary material for: Elevated expression of ANTXR1 gene in tumors is a poor prognostic biomarker for patients with bladder cancer
Source: Front Mol Biosci. 2025 Jan 23;11:1520223. doi: 10.3389/fmolb.2024.1520223 (PMC11798775; doi:10.3389/fmolb.2024.1520223)
Supplement: Supplementary file 1 [file DataSheet1.pdf]

Supplementary Figure 1. Workflow of the study

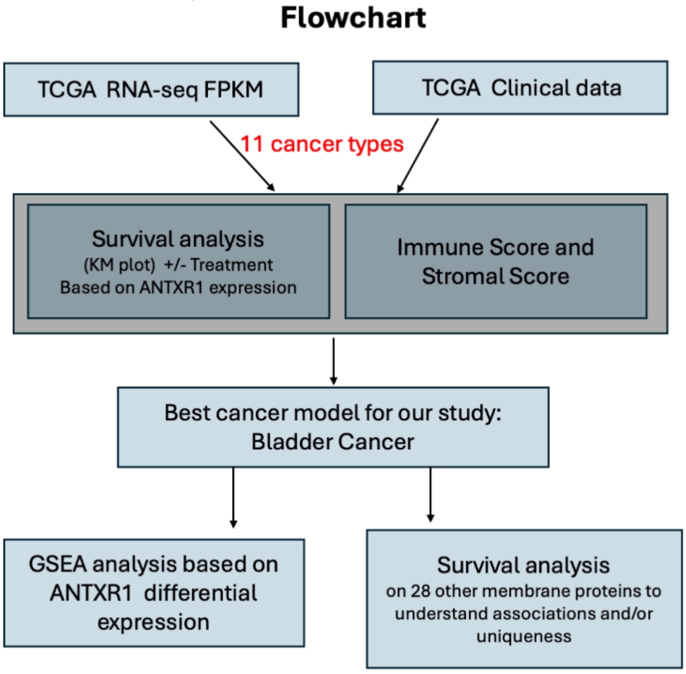

Supplementary Table 1. Downstream pathways in bladder cancer that are affected by TEM8 expression analyzed using MSigDB Hallmark. Highlighted pathways are congruent with pathways associated to cancer aggressiveness and progression.

	Pathway	FDR	NES
0	Oxidative Phosphorylation	0	-2.8697115
1	Coagulation	0	2.76274066
2	E2F Targets	0	-2.3312048
3	Myogenesis	0	3.0762079
4	Epithelial Mesenchymal Transition	0	4.07482639
5	Apical Junction	0	2.84508163
6	Myc Targets V2	0	-2.2336279
7	Myc Targets V1	0	-2.7139022
8	Inflammatory Response	0	2.72953587
9	UV Response Dn	0	2.70771618
10	Complement	0	2.50223448
11	Allograft Rejection	0	2.78170087
12	KRAS Signaling Up	0	2.50837133
13	Hedgehog Signaling	0.00071371	1.89125089
14	Hypoxia	0.00075832	1.94071736
15	TGF-beta Signaling	0.00080887	2.10038695
16	IL-6/JAK/STAT3 Signaling	0.00086665	2.2604092
17	TNF-alpha Signaling via NF-kB	0.00093331	2.33282817
18	IL-2/STAT5 Signaling	0.00101109	2.33515367
19	Interferon Gamma Response	0.00110301	2.38095996
20	Angiogenesis	0.00121331	2.41089117

FDR: False discovery rate; NES: Normalized enrichment score.
